# A study of staff׳s awareness and attitudes towards the importance of household hazardous wastes (HHW) management (A Case Study of Kermanshah University of Medical Sciences, Kermanshah, Iran)

**DOI:** 10.1016/j.dib.2018.06.022

**Published:** 2018-06-19

**Authors:** Yahya Safari, Kamaladdin Karimyan, Vinod Kumar Gupta, Arash Ziapour, Masoud Moradi, Nasrin Yoosefpour, Maliheh Akhlaghi, Hooshmand Sharafi

**Affiliations:** a Research Center for Environmental Determinants of Health (RCEDH), Kermanshah University of Medical Sciences, Kermanshah, Iran; b Environmental Health Research Center, Kurdistan University of Medical Sciences, Sanandaj, Iran; c Department of Environmental Health Engineering, Faculty of Public Health, Tehran University of Medical Sciences, Tehran, Iran; d Department of Applied Chemistry, University of Johannesburg, Johannesburg, South Africa; e Kermanshah University of Medical Sciences, Kermanshah, Iran; f Student research committee, Kermanshah University of Medical Sciences, Kermanshah, Iran; g Environmental Health Engineering, Health Faculty, Students Research Committee, Gonabad University of Medical Sciences, Gonabad, Iran

**Keywords:** Awareness, Attitude, HHWs, KUMS personnel, Waste management

## Abstract

The present study aimed to assess the levels of staff׳s awareness and attitudes towards the importance of household hazardous wastes (HHW) management at Kermanshah University of Medical Sciences (KUMS), Kermanshah, Iran. The awareness and attitudes were measured using a researcher-made questionnaire, which was then completed by 200 personnel at KUMS with different responsibilities. Finally, the data were then analyzed using the SPSS Statistical Software Version 21.0. The results of the present study showed that the average of awareness for man and woman was obtained 19.59 ± 3.53 and 19.88 ± 3.33, respectively. While, the attitude for them was 58.66 ± 9.5 and 61.25 ± 9.8, respectively. In terms of variable “job type”, the highest score about awareness and attitude was related to physician (20.45 ± 2.41) and nurse (61.8 ± 9.2) jobs respectively. The highest level of awareness was for those with a diploma degree, while in term of attitude the maximum score was obtained for those who were undergraduate and bachelor degree. Based on age group, minimum and maximum score of awareness were related to 44–53 and 44–53 years, respectively. While in term of attitude were 54–65 and 34–43 years, respectively. According to results, it is suggested that households be trained in separating, recycling, collecting, transporting and disposing of HHWs in accordance with health standards with the aim of providing, maintaining and improving the health of families, societies and environment. It should be noted that prevention of adverse environmental effects of hazardous waste is a priority, which can be realized through applying proper management methods.

**Specifications Table**

**Table:** 

Subject Area	Environmental Sciences
More specific subject area	Environmental Health
Type of Data	Tables
Data Collection Method	To conduct the present, a researcher-made questionnaire was first designed to measure the level of awareness and attitude of 200 staff at Kermanshah University of Medical Sciences (KUMS) with different responsibilities about HHWs. Further, the data were then analyzed using the SPSS Statistical Software Version 21.0.
Data Format	Raw, analyzed
Experimental factors	The validity and reliability of the questionnaire were evaluated using content validity and Cronbach׳s alpha.
Experimental Features	To compare the means of two groups of variables and more, the independent sample t-test and ANOVA were used respectively.
Location of Data Source	Kermanshah City, Iran
Data Accessibility	Data were included in this article

**Value of the data**

• HHWs account for only 1% of the total municipal solid waste. However, this low level can still pose many risks to the environment and human health [1], [2], [3], [4]. Moreover, the data from the present study is about evaluating the awareness and attitudes of people towards the above-mentioned risks.
• Limited studies have been conducted on the subject under discussion. Therefore, the data from present study can serve as the basis for future studies. In addition, due to the lack of previous studies in this respect, the obtained data is useful for understanding the status of awareness and attitudes of people about HHWs.
• The management style of HHWs produced in its primary location (personal home) can be affected by the final treatment of domestic wastewater and the final disposal of municipal solid waste [5], [6], [7], [8], [9], [10], [11], [12]. Therefore, it is very important to measure and promote the awareness and attitudes of people about HHWs.
• The data from the present study can serve as a basis for future studies about HHWs in Iran, especially in the area under study, i.e., Kermanshah.

## 1 Data

According to the demographic data of the subjects under study, men accounted for slightly above half of the sample population (51% or 102 subjects), and the rest were women (49% or 89 subjects). Moreover, the married subjects made up about 61.5% (123 subjects) of the sample population, whereas the rest were single (38.5% or 77 subjects). Besides, 57% (11 subjects) were living in bungalows, and the rest were in apartments (42.5% or 85 subjects) (Table 1). The results revealed that the awareness of the subjects under study was high in terms of two components, and very high in terms of two other components (Table 2). As for the attitude of subjects under study, the results indicated that it was high in terms of two components, and very high in terms of three other components (Table 3). Additionally, there were significant differences between the mean scores of awareness of subjects in terms of marital status, job, and age group (*P* < 0.05) whereas no significant difference was observed for other variables (*P* > 0.05). Furthermore, there were significant differences between the mean scores of attitudes of subjects in terms of gender, type of residence and academic degree (*P* < 0.05) whereas no significant difference was observed for other variables (*P* > 0.05) (Table 4).

**Table 1:** The demographic data of the subjects under study.

**Variables**		**Frequency**	
		**Number**	**Percent**
Sex	Man	102	51
	Woman	98	49
Type of residential home	Villa׳s house	115	57.5
	Apartment house	85	42.5
Marital status	Married	123	61.5
	Single	77	38.5
Job	University faculty member	51	25.5
	Physician	49	24.5
	Nurse	49	24.5
	Administrative officer	51	25.5
Education level	Elementary and middle school	10	5
	Diploma	20	10
	Undergraduate and Bachelor	75	37.5
	MA and Ph.D.	95	47.5
Age group (years)	23–33	81	40.5
	34–43	63	31.5
	44–53	46	23
	54–65	10	5
	Total	200	100

**Table 2:** The scores obtained by the subjects on awareness based on sub-goals and likert scale.

**Knowledge**		
**Special goals**	**Acquired scores (Max)**	**Knowledge level**
1. Awareness of various hazardous household waste materials and their properties	4.08(5)	Very good
2. Awareness of the health and environmental effects of hazardous household waste	6.39(8)	Good
3. Awareness of the adverse effects of hazardous waste management on the proper management of normal household waste	5.16(7)	Good
4. Awareness of the proper management (collection, processing and disposal) of hazardous household waste	4.09(5)	Very good

**Table 3:** The scores obtained by the subjects on attitude based on sub-goals and likert scale.

**Attitude**		
**Special goals**	**Acquired scores (Max)**	**Attitude level**
1. The importance of considering hazardous household waste materials and their properties	7.63(9)	Very good
2. The importance of the health and environmental effects of hazardous household waste	20.48(24)	Very good
3. The importance of the adverse effects of hazardous waste management on the proper management of normal household waste	12.165(15)	Very good
4. The importance the proper management (collection, processing and disposal) of hazardous household waste	12.91(18)	Good
5. The importance of media training in the management of household hazardous wastes	6.74(9)	Good

**Table 4:** The total scores obtained by the subjects on awareness and attitude based on the variables under study.

**Variables**		**Overall awareness**		**Overall attitude**	
		**Mean ± SD**	**P**	**Mean ± SD**	**P**
Sex	Man	19.59 ± 3.53	0.551	58.66 ± 9.5	0.04
	Woman	19.88 ± 3.33		61.25 ± 9.8	
Type of residential home	Villa׳s house	19.39 ± 4.04	0.041	60.05 ± 11.1	0.82
	Apartment house	20.27 ± 1.94		59.7 ± 7	
Marital status	Married	19.41 ± 3.94	0.054	58.2 ± 10.3	0.002
	Single	20.02 ± 2.47		62.34 ± 8.55	
Job	University faculty member	19.22 ± 1.48	0.309	56.8 ± 12.2	0.036
	Physician	20.45 ± 2.41		61.57 ± 7.56	
	Nurse	20.13 ± 2.65		61.8 ± 9.2	
	Administrative officer	19.31 ± 4.14		59.7 ± 8.9	
Education level	Elementary and middle school	18.06 ± 5.15	0.001	56.44 ± 11.78	0.053
	Diploma	20.69 ± 1.71		60.4 ± 9.2	
	Undergraduate and Bachelor	20.53 ± 2.63		61.13 ± 8.6	
	MA and Ph.D.	19.71 ± 2.44		58.9 ± 10.54	
Age group (years)	23–33	20.36 ± 2.4	0.016	60.52 ± 7.1	0.302
	34–43	20.00 ± 2.99		60.7 ± 10.8	
	44–53	18.47 ± 4.30		58.9 ± 10.7	
	54–65	18.90 ± 6.11		55 ± 15.5	

## 2 Study Design, Materials and Methods

To carry out the present experimental study, a researcher-made questionnaire was first designed to measure the level of awareness and attitudes of 200 staff at Kermanshah University of Medical Sciences (KUMS) with different responsibilities about HHWs. To this end, the basic principles of municipal solid waste and HHWs were reviewed in books and articles [7], [8], [9]. The meaning of "waste" In the phrase "hazardous household waste" is both hazardous liquid waste and hazardous solid waste.

The validity of the questionnaire was evaluated using content validity. To do so, the intended questionnaire was given to 10 faculty members of the Faculty of Health of KUMS and 10 employees at the environmental health centers of Kermanshah to be examined based on the objectives of the study and the questions relating to attitude and awareness. Furthermore, the reliability of the questionnaire was evaluated using Cronbach׳s alpha (α=0.87) [13], [14], [15]. To conduct the present study, 200 subjects were selected as the final sample, who were extracted from a statistical population of 2500 people using the Morgan Sample Size Table and previous studies. In addition, according to the share of each group (cluster sampling), 40 physicians and specialists who were not faculty members, 56 faculty members, 44 nurses and 60 administrative staff were selected randomly by simple random sampling method.

Given that each subsidiary target group was covered by various questions of the questionnaire, the Likert range of each target group was different in terms of score (Table 5, Table 6). After filling out the awareness and attitude questionnaires, the obtained results were transferred to the SPSS Statistical Software Version 21.0. Moreover, to compare the means of two groups of variables and more, the independent sample t-test and ANOVA were used respectively. Finally, the descriptive parameters were presented using the descriptive statistics.

**Table 5:** The rankings of awareness levels for each of the specific goals based on the likert scale.

**Special goals**	**Maximum score**	**Knowledge level**			
		**Very good**	**Good**	**Medium**	**Weak**
1. Awareness of various hazardous household waste materials and their properties	5	0–1.25	1.25–2.5	2.5–3.75	3.75–5
2. Awareness of the health and environmental effects of hazardous household waste	8	0–2	2–4	4–6	6–8
3. Awareness of the adverse effects of hazardous waste management on the proper management of normal household waste	7	0–1.75	1.75–3.5	3.5–5.25	5.25–7
4. Awareness of the proper management (collection, processing and disposal) of hazardous household waste	5	0–1.25	1.25–2.5	2.5–3.75	3.75–5
**Overall knowledge**	25	0–6.25	6.25–12.5	12.5–18.75	18.75–25

**Table 6:** The rankings of attitude levels for each of the specific goals based on the likert scale.

**Special goals**	**Maximum score**	**Attitude level**			
		**Very good**	**Medium**	**Good**	**Very good**
1. The importance of considering hazardous household waste materials and their properties	9	0–2.25	2.25–4.5	4.5–6.75	6.75–9
2. The importance of the health and environmental effects of hazardous household waste	24	0–6	6–12	12–18	18–24
3. The importance of the adverse effects of hazardous waste management on the proper management of normal household waste	15	0–375	3.75–7.5	7.5–11.25	11.25–15
4. The importance the proper management (collection, processing and disposal) of hazardous household waste	18	0–4.5	4.5–9	9–13.5	13.5–18
5. The importance of media training in the management of household hazardous wastes	9	0–2.25	2.25–4.5	4.5–6.75	6.75–9
**Overall attitude**	75	0–18.75	18.75–37.5	37.5–56.25	56.25–75
