# Supplementary material for: A study of staff׳s awareness and attitudes towards the importance of household hazardous wastes (HHW) management (A Case Study of Kermanshah University of Medical Sciences, Kermanshah, Iran)
Source: Data Brief. 2018 Jun 19;19:1490–7. doi: 10.1016/j.dib.2018.06.022 (PMC6141165; doi:10.1016/j.dib.2018.06.022)
Supplement: Supplementary file 1 — Supplementary material [file mmc1.docx]

Dear editor(s)

Data in Brief

Greetings!

At this time, this article with entitled” ***Data on knowledge and attitude of Kermanshah University of medical sciences personnel on the importance of household hazardous wastes management*** “has not been published or submitted in any other journals. We hope that the results of this research will be of interest to you. None of the authors of this paper have any conflicts of interest to declare.

Sincerely yours
